# Effects of a postbiotic, with and without a saponin-based product, on turkey performance

**DOI:** 10.1016/j.psj.2023.102607

**Published:** 2023-02-21

**Authors:** Evan Chaney, Elizabeth A. Miller, Jeffrey Firman, Andrea Binnebose, Vivek Kuttappan, Timothy J. Johnson

**Affiliations:** ⁎ Diamond V, Cargill Health Technologies, Cedar Rapids, IA 52404, USA; † Department of Veterinary and Biomedical Sciences, University of Minnesota, Saint Paul, MN, USA; ‡ Missouri Contract Poultry Research, Boonville, MO 65233, USA

**Keywords:** postbiotic, saponin, poultry, turkey, performance

## Abstract

Modern poultry production relies on an ability to prevent and mitigate challenges to bird health, while maintaining a productive bird. A number of different classes of biologics-based feed additives exist, and many have been tested individually for their impacts on poultry health and performance. Fewer studies have examined the combinations of different classes of products. In this study, we examined the use of a well-established postbiotic feed additive (Original XPC, Diamond V) on turkey performance, with and without the addition of a proprietary saponin-based feed additive. This was accomplished in an 18-wk pen trial utilizing 22 pen replicates per treatment across 3 treatments (control, postbiotic, and postbiotic plus saponin). Significant differences in body weight were identified at wk 12 and 15 of age, with the postbiotic plus saponin treatment group resulting in heavier birds at both timepoints. Significant differences in feed conversion ratio were observed from 0 to 18 wk of age, with the postbiotic alone having improved FCR compared with the control group. No significant differences were observed for livability or feed intake. This study demonstrates that a combination of a postbiotic plus saponin may exert additive effects on the growth of the turkey.

## INTRODUCTION

Many challenges face poultry producers, and each of these challenges has the potential to reduce the performance and overall health of the flock. Challenges include those which are environmental, microbial, developmental, and nutritional, among others. Each of these challenges alone may have a negative impact on performance and health, but can be exacerbated when they are combined. From a microbial standpoint, defining threats is quite difficult because many biological threats are also “normal” members of the microbial consortia inhabiting the birds. These normal members become a threat when there is sufficient stress, and the threshold for this threat is difficult to define.

Broadly, biologics-based feed additives can be classified as prebiotics, probiotics, and postbiotics (Zolkiewicz et al., 2020). Prebiotics are substrates which are selectively utilized by host microorganisms conferring a health benefit (Gibson et al., 2017). Prebiotics are not typically digested by the host, but they feed both the indigenous microbiota, and microorganisms exogenously supplied to the host. In contrast, probiotics are live microorganisms that, when administered in the appropriate place/time and in adequate amounts, confer a health benefit on the host (Hill et al., 2014). Both prebiotics and probiotics have the potential to modulate the natural microbiota, but they also may have an array of positive indirect impacts on the host. The use of prebiotics and probiotics in combination are referred to as synbiotics, with the implication that such approaches have synergistic effects on the host. Postbiotics are defined as any preparation of inanimate or inactivated microorganisms and their components that confer a health benefit on the host (Salminen et al., 2021). These include materials such as cell-free supernatants, specific metabolites, biopolymers released outside of the cell wall, and specific enzymes. Postbiotics are a relatively newly named class of products, although they have been around for some time already.

Original XPC (Diamond V, Cedar Rapids, IA) is a postbiotic feed additive produced as a proprietary *Saccharomyces cerevisiae* fermentation product (**SCFP**). Dietary inclusion of XPC has been associated with fat digestibility improvements in laying hens (Tonkinson et al., 1965) and performance benefits in a variety of other production animal species (Price et al., 2010; Poppy et al., 2012; Pinheiro et al., 2017). In poultry species, inclusion of SCFP into dietary rations have resulted in improvements in key areas including immunity, gut health and tissue integrity, microbiota modulation, and production performance (Gao et al., 2008, 2009; Lensing et al., 2012; Park et al., 2017; Nelson et al., 2018; Price et al., 2018). Published literature reporting SCFP effects in turkeys, specifically, has been relatively limited. However, benefits have been observed in turkey embryonic mortality and hatchability when breeder hens were fed SCFP (Bradley, 1995); in body weights and FCR when turkey progeny and their breeder parents were fed SCFP (Ferket, 2011); and in FCR and breast meat yield when male turkeys were fed SCFP (Firman et al., 2013). Further research is warranted to evaluate the effects and benefits of feeding SCFP postbiotic alone or in combination with additional products on the performance and health benefits of commercial turkeys.

Phytogenic feed additives have great potential, but only few studies have evaluated the synergy between phytogenic and postbiotics. Specific to combining yeast-based products with saponin-containing products, few studies have examined their effects. One study examining dietary supplementation of yeast cell walls with *Yucca schidigera* extract found that the combination numerically improved growth performance and feed efficiency compared to either product alone (Gurbuz et al., 2011). A second study compared *Yucca schidigera* extract and *Saccharomyces boulardii* effects on broiler performance individually, but not in combination (Preston et al., 1999). A number of studies have examined the effects of yeast-based products on commercial turkey performance (Bradley et al., 1994; Huff et al., 2013; Shanmugasundaram et al., 2014; Rahimi et al., 2019; Lipinski et al., 2021), but no published studies to our knowledge have examined the addition of a saponin-based product, to diets including a yeast-based product, on turkey performance. Therefore, the goal of the present study was to examine the effects of this combination.

## MATERIALS AND METHODS

### Bird Housing and Care

Day of hatch Nicholas/Hybrid male poults (*N* = 858, 13 per pen) were obtained from a commercial hatchery. No other vaccinations were provided. Poults were allocated to 1 of 66 pens. Twenty-two replicate pens were assigned per treatment with 13 birds per pen (*n* = 286 birds per treatment). The pen facility was divided into 6 blocks of 11 pens, with treatments assigned to pens using randomized complete block design. Randomization and assignment of treatments to pens was performed using a random number generator.

Birds were housed in an industry-standard tunnel ventilated house on used litter with an anticoccidial (Coban) added to the feed at 0.05% (1.0 lb/ton). The purpose of used litter was to provide additional microbial challenges to the birds. Pens measured 7’ × 8’ feet minus feeder/waterer space. Temperature and ventilation followed standard industry practices, with a starting temperature of 31°C lowered by approximately 3°C per wk using daily incremental computer controls, adjusted for bird comfort. Feed and water were provided ad libitum throughout the trial using one tube feeder and Plasson drinker in each pen. The house received 24 h of light for the first 7 d, and 16 h of light and 8 h of darkness until study completion at d 126. Lights were dimmed to approximately 0.5 fc at d 8. Birds were raised under University of Missouri Animal Care and Use Committee Exempt Protocol #9851.

### Treatments and Performance Measurements

Three diets were included in this study. The control diet (referred to as Control) was a standard multiphase basal turkey diet. The diet was a standard corn-soybean diet fed in 6 phases (0–3 wk, 3–6 wk, 6–9 wk, 9–12 wk, 12–15 wk, and 15–18 wk). Corn:soybean meal was started at 43.5:41.5% and finished at 68.6:15.7%. Crude protein content started at 26.0% and was dropped in each phase through a finishing content of 16.0%.

Two additional experimental diets were included. One of these included Diamond V Original XPC (referred to as XPC), a proprietary *Saccharomyces cerevisiae* fermentation product, added to the basal diet at an inclusion rate of 1.25 kg/MT (2.5 lb/t) throughout all phases. The second experimental diet also included Original XPC added to the basal diet at an inclusion rate of 1.25 kg/MT (2.5 lb/t), plus the addition of a proprietary saponin-based product added at 0.25 kg/MT (0.5 lb/t), throughout all phases (Poultry Plus, referred to as XPC+). Feed was prepared at the University of Missouri Research Feed Mill. Diets were blinded to researchers using letters for treatments. Samples of prepared feed were retained, and tested to confirm dietary inclusions using a unique iron micro-tracer for each diet, coated with a different coloring agent.

Performance metrics measured included body weights at time of placement and wk 6, 9, 12, 15, and 18. Weights were taken at the pen level (total pen weight divided by number of birds) through wk 9 of age, and at the individual bird for the remainder of the study. Mean individual body weights per pen were used for all analyses. Weight gains were calculated for each time interval by subtracting initial from final pen-level weights, correcting for mortality weights and days of feeding lost for each bird, and averaging per bird based on number of birds in each pen at the start and completion of the interval. Feed was weighed back at the pen level at each of these timepoints to determine feed consumed per pen during the time interval. Mortality-adjusted feed conversion ratios were then calculated using feed consumed to weight gained at the individual bird level, also correcting for mortality weights and days lost. Mortality was assessed daily, and presented weekly as livability, or percentage of birds surviving at that timepoint relative to starting numbers in each pen.

### Statistical Analyses

All statistics were run in R (v.4.1.2). Separate regression models were constructed for each metric at each time point/period. For all metrics (except livability) we used linear mixed-effect models with function lmer() from R package *lme4* (v.1.1-27.1). Treatment was considered as an independent variable. Body weights at time zero were included as a covariate in all body weight models for wk 6 to 18 of age. Block was included as a random intercept. We tested the overall importance of Treatment by running a type II ANOVA with Satterthwaite's method. Post hoc pairwise comparisons between all 3 treatment groups were achieved by calculating the estimated marginal means (least-squares means) using the R package *emmeans* (v.1.7.4-1) with a Tukey method correction for multiple testing. Differences were considered significant when *P* < 0.05. For livability, a generalized linear mixed model was used with a binomial error structure and logit link function. For livability models, *P* values were calculated using type II Wald chi-square tests with the Anova() function from the *car* package (v.3.0-12). Post hoc pairwise comparisons between all 3 treatment groups were achieved by calculating the estimated marginal means (least-squares means) using the R package *emmeans* (v.1.7.4-1) with a Tukey method correction for multiple testing. Differences were considered significant when *P* < 0.05.

## RESULTS AND DISCUSSION

There was a near linear increase in body weights over the time periods assessed. Assessing mean individual body weights with pen as the experimental unit, and accounting for time zero weights, significant differences were observed at wk 12 (*P* = 0.03) and 15 (*P* = 0.048) (Table 1). At wk 12 of age, XPC+ weights were significantly higher than the Control group (pairwise *P* = 0.02), and the XPC group was a statistical intermediate. At wk 15 of age, XPC+ weights were significantly (*P* = 0.048) higher than the Control group and XPC . While not statistically significant, at wk 18 of age, the XPC and XPC+ treatment groups were +0.36 and +0.46 kg (+0.79 and +1.01 lbs) compared to Control, respectively. There was also a numerical trend at all observed timepoints of higher body weights in XPC and XPC+ treatment groups, compared to the Control group.

**Table 1:** Mean turkey body weights (kg) and CV (%) by week of age and treatment.

		Week of age					
		0	6	9	12	15	18
Mean body weight (kg)	Control	0.06 ± 0.00	2.63 ± 0.03	5.97 ± 0.06	9.77 ± 0.09^b^	13.93 ± 0.13^b^	16.60 ± 0.14
	XPC	0.06 ± 0.00	2.63 ± 0.03	6.07 ± 0.07	9.92 ± 0.09^ab^	14.10 ± 0.14^b^	16.96 ± 0.15
	XPC+	0.06 ± 0.00	2.70 ± 0.03	6.15 ± 0.06	10.10 ± 0.09^a^	14.41 ± 0.14^a^	17.06 ± 0.14
	*P* value	0.20	0.27	0.13	0.03	0.048	0.06
Coefficient of variation (CV; %)	Control	1.35	4.24	4.68	4.50	5.41	3.47
	XPC	0.97	7.62	5.79	4.85	4.33	4.61
	XPC+	1.49	6.15	4.26	3.90	3.67	4.97

Superscript letters indicate statistical signficance between treatment groups (*P* < 0.05).

**Figure 1:**
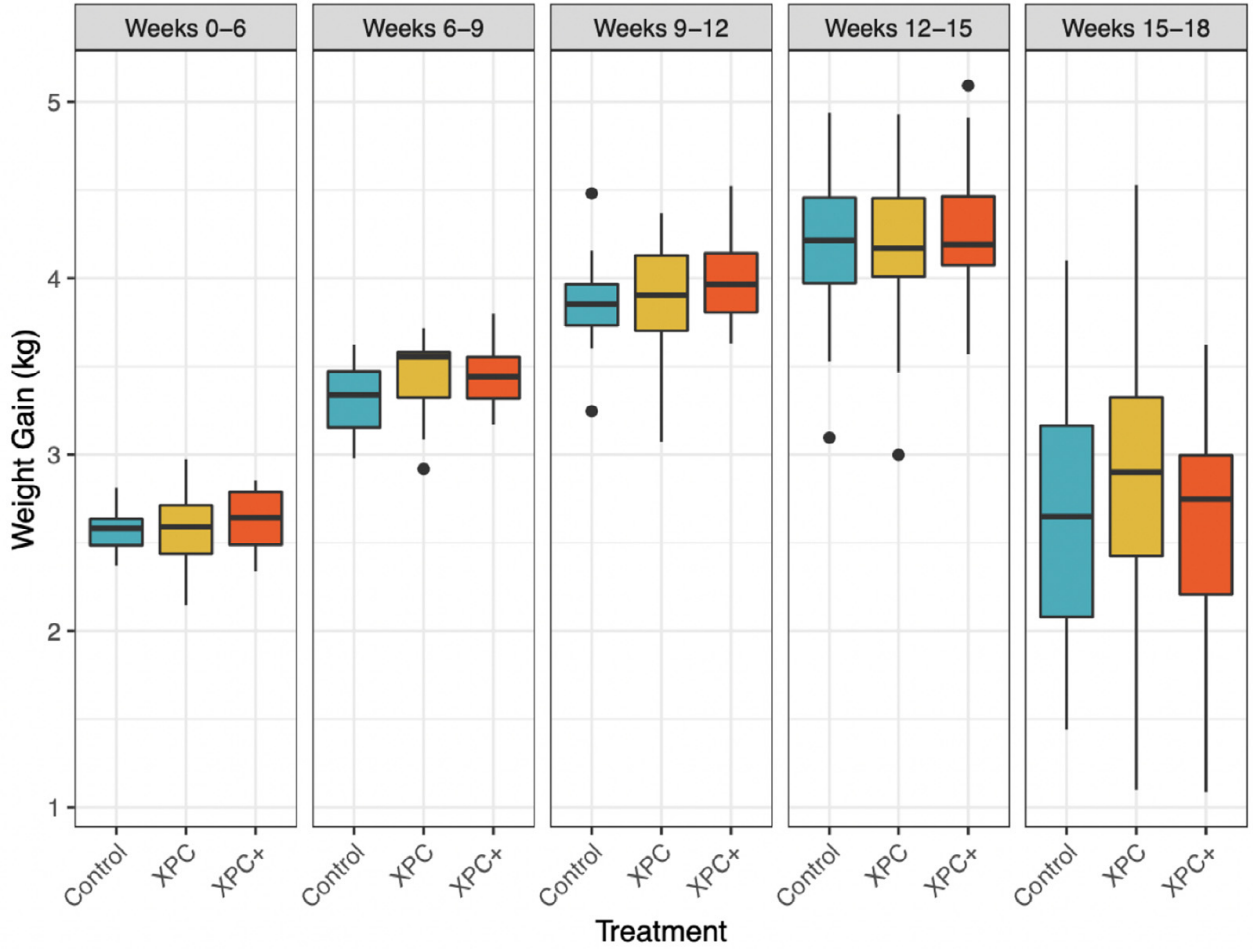
Average weight gain per turkey during intervals of measurement in this study, grouped by treatment.

Rate of weight gain was assessed during specific time intervals throughout this study (Figure 1). Rate of gain increased over time until wk 15 to 18, where it dropped substantially. There were no significant differences between treatment groups at any of the time intervals assessed (data not shown). However, there were some numerical increases in rate of gain for the XPC and XPC+ treatment groups compared to the Control group at respective timepoints. Cumulatively, from wk 0 to 12 there were significant differences between treatment groups in weight gain (*P* = 0.03), where the XPC+ treatment group had significantly higher gain than the Control group (*P* = 0.02) and the XPC group was a statistical intermediate.

At 18 wk of age, the XPC treatment group had significantly (*P* < 0.05) improved FCR compared to the Control group, and the XPC+ group was a statistical intermediate. The XPC and XPC+ treatment groups had 8.1 and 4.3 points improved FCR compared to the Control group, respectively. At all timepoints, the XPC and XPC+ groups had numerically improved FCR values compared to the Control group (Table 2).

**Table 2:** Livability, feed conversion ratios (FCR), and feed intake by time interval.

		Week of age				
		Wk 0–6	Wk 0–9	Wk 0–12	Wk 0–15	Wk 0–18
Livability (% pen)	Control	100.0 ± 0.0	98.0 ± 0.1	96.5 ± 1.1	95.5 ± 1.2	89.9 ± 1.8
	XPC	100.0 ± 0.0	98.3 ± 0.1	95.8 ± 1.2	93.0 ± 1.5	90.2 ± 1.8
	XPC+	100.0 ± 0.0	99.7 ± 0.3	99.3 ± 0.5	97.2 ± 1.0	93.4 ± 1.5
	*P* value	NA	0.19	0.06	0.07	0.27
FCR (feed:gain)						
	Control	1.631 ± 0.019	1.690 ± 0.01	1.868 ± 0.011	2.105 ± 0.016	2.357 ± 0.019^a^
	XPC	1.615 ± 0.018	1.670 ± 0.01	1.857 ± 0.012	2.071 ± 0.016	2.276 ± 0.020^b^
	XPC+	1.602 ± 0.018	1.666 ± 0.01	1.831 ± 0.011	2.061 ± 0.016	2.314 ± 0.018^ab^
	*P* value	0.55	0.20	0.07	0.13	0.01
Feed intake (kg per bird)	Control	4.11 ± 0.06	9.91 ± 0.13	18.03 ± 0.22	28.68 ± 0.34	38.01 ± 0.44
	XPC	4.06 ± 0.06	9.77 ± 0.13	17.92 ± 0.22	28.64 ± 0.34	37.88 ± 0.43
	XPC+	4.19 ± 0.06	10.10 ± 0.13	18.26 ± 0.21	28.92 ± 0.34	38.10 ± 0.43
	*P* value	0.3264	0.1909	0.5037	0.813	0.9323

Superscript letters indicate statistical signficance between treatment groups (*P* < 0.05).

No statistically significant differences between treatment groups were observed at any time interval for livability, which was defined as percent of birds surviving in a pen during a time interval compared with starting bird numbers for that respective pen (Table 2). Similarly, no statistically significant differences between treatment groups were observed at any time interval for cumulative feed intake per bird.

The greatest enhancement of body weights compared to the Control group were observed at wk 12 and 15 of age (+3.4% differential in XPC+ vs. Control), and this was also reflected by weight gain where the strongest differentials in gain were observed from wk 6 to 9 and 0 to 12 in the study. Subjectively, these periods of the study were ideal conditions for raising turkeys based on ambient temperature and humidity. During wk 15 to 18 of the study, ambient temperature and humidity were elevated and it appeared that growth of the birds slowed. Presumably, birds were under stress during this period. During this same period, weight gain and FCR were numerically improved in the XPC group compared to both Control and XPC+ groups. This does suggest that there may be some trade-offs for using such combinations of products during certain production conditions. However, these differences were not statistically significant, therefore further work is needed to fully understand these product interactions.

In conclusion, the current study evaluated the benefits of feeding a postbiotic-product with or without saponin in turkeys. Results showed that inclusion of XPC in diet improved body weight either numerically or significantly throughout the grow out period compared to CON. Interestingly, the XPC fed birds had significantly better FCR at the end of the grow out compared to CON. Inclusion of saponin along with XPC showed some additional benefits in improving body weight, but further studies are warranted to explore the benefits of the combination.
